# Clinical Correlates of Early-Onset Hypertension

**DOI:** 10.1093/ajh/hpab066

**Published:** 2021-04-27

**Authors:** Karri Suvila, Joao A C Lima, Susan Cheng, Teemu J Niiranen

**Affiliations:** 1 Division of Medicine, Turku University Hospital and University of Turku, Turku, Finland; 2 Division of Cardiology, Johns Hopkins University, Baltimore, Maryland, USA; 3 Division of Cardiovascular Medicine, Department of Medicine, Brigham and Women’s Hospital, Boston, Massachusetts, USA; 4 Department of Cardiology, Smidt Heart Institute, Cedars-Sinai Medical Center, Los Angeles, California, USA; 5 Framingham Heart Study, Framingham, Massachusetts, USA; 6 Department of Public Health Solutions, Finnish Institute for Health and Welfare, Turku, Finland

**Keywords:** age of onset, blood pressure, epidemiology, hypertension, risk factors

## Abstract

**BACKGROUND:**

Early-onset hypertension has been established as a heritable trait and a risk factor for cardiovascular disease outcomes. However, the clinical correlates of early-onset hypertension remain unidentified.

**METHODS:**

In this study, we assessed the demographic characteristics and lifestyle factors related to hypertension onset age in a sample of 3,286 Coronary Artery Risk Development in Young Adults (CARDIA) study participants (mean baseline age 25 ± 4 years, 57% women). We examined the association between the participants’ baseline characteristics and age of hypertension onset subgroups (<35, 35‒44, or ≥45 years) using a multinomial logistic regression model with those who did not develop hypertension as the reference group. Hypertension onset was defined as blood pressure ≥140/90 mm Hg or antihypertensive medication use on 2 consecutively attended follow-up visits.

**RESULTS:**

In the multinomial logistic regression model, individuals who were black (odds ratio [OR], 5.08; 95% confidence interval [CI], 3.17–8.14), were more obese (OR, 1.57; 95% CI, 1.32–1.88), or had higher total cholesterol (OR, 1.34; 95% CI, 1.13–1.60 per SD) had increased odds of early-onset hypertension (onset at <35 years) vs. not developing hypertension. In contrast, 1-SD higher high-density lipoprotein (HDL)-cholesterol was related to decreased odds of early-onset hypertension (OR, 0.71; 95% CI, 0.57–0.89). The odds for having earlier hypertension onset increased linearly across age of onset categories in black individuals and individuals with lower HDL-cholesterol (*P* < 0.05 for trend for both).

**CONCLUSIONS:**

Our findings suggest that individuals who are black, obese, have higher total cholesterol, or have lower HDL-cholesterol level, are potentially at an increased risk of having early-onset hypertension.

## Graphical Abstract

**Graphical Abstract:**
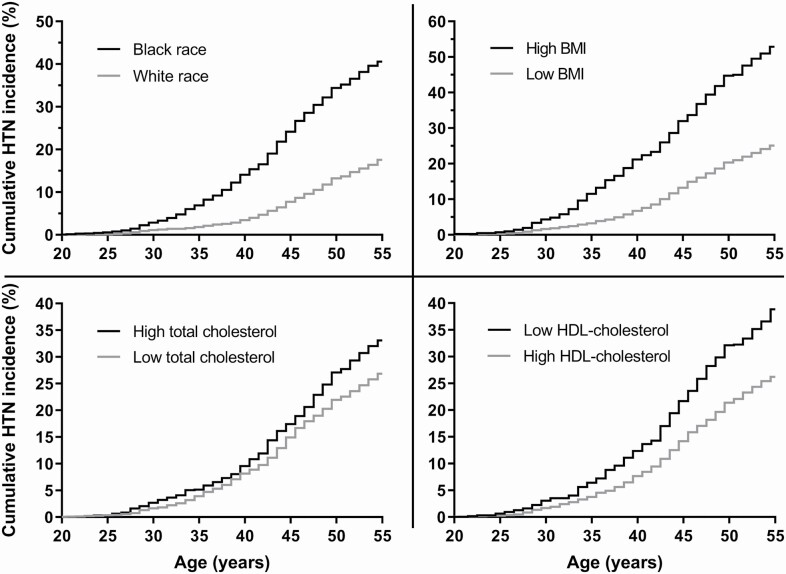

Early-onset hypertension is a familial trait that confers increased risk for hypertensive organ damage and cardiovascular death compared with late-onset hypertension.^1–3^ Only few studies in highly selected study samples have assessed common characteristics in individuals with early-onset hypertension.^4,5^ However, the clinical correlates of early-onset hypertension, which could be used for assessing disease risk, remain unknown. Our objective was therefore to evaluate the demographic characteristics and lifestyle factors related to age of hypertension onset in CARDIA (Coronary Artery Risk Development in Young Adults) study participants. We hypothesized that specific clinical characteristics would associate with having early-onset hypertension.

## METHODS

The initial cohort included 5,115 individuals aged 18–30 years with approximately equal number of participants by sex, race, and education.^6^ The data underlying this article are based on the CARDIA study and are available at the National Institutes of Health’s Biologic Specimen and Data Repository Information Coordinating Center and can be accessed at https://biolincc.nhlbi.nih.gov/studies/cardia/. The participants attended up to 9 follow-up examinations conducted at regular intervals between 1985–1986 and 2015–2016. We included only participants who attended the Year 30 examination (*n* = 3,358) so that all participants could be assigned to all hypertension onset age categories. After excluding participants with missing baseline data, the final sample consisted of 3,286 individuals (mean age 25 ± 4 years at baseline; 57% women). The study was approved by local institutional review boards and all participants provided informed consent.

Blood pressure was measured at all follow-up visits by using a mercury sphygmomanometer during the first 6 examinations and an oscillometric device at Years 20, 25, and 30.^2,6^ The oscillometric measures were calibrated to sphygmomanometer values by using a previously introduced formula.^7^ Hypertension onset was defined as blood pressure ≥140/90 mm Hg or antihypertensive medication use on 2 consecutively attended examinations.^2^ Individuals who did not meet the criteria for hypertension onset were considered normotensive. The participants were divided into 4 categories: hypertension onset at age <35 years (*n* = 122; early-onset hypertension), onset between 35‒44 years (*n* = 332), onset at ≥45 years (*n* = 486), or normotension (*n* = 2,346). Information on demographic characteristics, smoking, education years, alcohol intake, and medication use was collected with self-administered questionnaires. Body mass index (BMI) was calculated from measured height and weight. Total cholesterol, high-density lipoprotein (HDL)-cholesterol, and glucose levels were quantified from fasting blood samples.^2,6^ We defined diabetes as serum fasting glucose of ≥7 mmol/l or antihyperglycemics use. As the prevalence of diabetes was <0.5% at baseline, we did not include it in the analyses.

We studied the baseline characteristics of the entire study sample and in subgroups by hypertension onset age. We also compared the participants characteristics using a χ ^2^ test for categorical variables and analysis of variance for continuous variables. We tested for trend across hypertension onset age categories using the Cochran–Armitage trend test for categorical variables and linear regression for continuous variables. We evaluated the association between the participants’ baseline characteristics and hypertension onset age subgroups using a multinomial logistic regression model. All traits were simultaneously included in the model with the age of hypertension onset category (<35, 35‒44, and ≥45 years) as the dependent variable with those who did not develop hypertension as the reference group. We also tested for a trend in odds ratios across hypertension onset age categories using linear regression. Continuous variables were standardized into *z*-scores for analyses. Clinical characteristics in the model included sex (male vs. female), race (black vs. white), BMI (kg/m^2^), smoking (current smoker vs. no smoking), education (years), alcohol use (ml/day), total cholesterol (mmol/l), and HDL-cholesterol (mmol/l). We considered 2-tailed *P* < 0.05 as statistically significant.

## RESULTS

Overall, 28.6% (*n* = 940) of the participants had experienced hypertension onset by the Year 30 examination. Detailed characteristics of the study sample and by age of hypertension onset subgroups are presented in Supplementary Table S1 online. Briefly, in the early-onset hypertension group (onset at age <35 years), 51% were women, 78% were black, and 20.5% currently smoked. We observed an increasing trend across the 4 hypertension onset age categories for black race, smoking, BMI, total cholesterol, and blood pressure (*P* < 0.01 for difference across groups). Conversely, a decreasing trend was detected for education years and HDL-cholesterol (*P* < 0.001 for difference across groups).

In the multivariable multinomial logistic regression model, individuals who were black, had higher BMI, or had higher serum total cholesterol level had increased odds for having early-onset hypertension (onset at age <35 years) compared with those without hypertension (Table 1). Conversely, higher serum HDL-cholesterol level was related to decreased odds for having early-onset hypertension. The same traits, expect for total cholesterol, were also associated with other hypertension onset age categories (*P* < 0.05 for all). The odds for having earlier hypertension onset increased linearly across age of onset categories in black individuals (*P* < 0.001 for trend), while the odds decreased linearly according to higher serum HDL-cholesterol (*P* < 0.05 for trend). Additionally, male sex was associated with decreased odds of hypertension onset at ≥45 years. In contrast, participants with higher education had decreased odds of hypertension onset between 35 and 44 years of age (*P* < 0.01). Alcohol intake was not related to hypertension onset age (*P* > 0.41 for all).

**Table 1.** Odds of hypertension onset age categories according to the CARDIA study participants clinical characteristics

	Odds ratios (95% confidence intervals) for hypertension onset				
Characteristic	<35 years	35–44 years	≥45 years	No HTN	Trend in ORs (*P* value)
Male sex	1.09 (0.73–1.61)	0.82 (0.63–1.06)	0.80 (0.65–1.00)^‡^	1.00	0.79
Black race	5.08 (3.17–8.14)*	3.63 (2.73–4.83)*	2.28 (1.82–2.85)*	1.00	<0.001
BMI	1.57 (1.32–1.88)*	1.52 (1.35–1.72)*	1.47 (1.32–1.64)*	1.00	0.14
Smoking	1.28 (0.83–1.98)	0.93 (0.70–1.24)	1.46 (1.15–1.84)^†^	1.00	0.83
HDL-cholesterol	0.71 (0.57–0.89)^†^	0.84 (0.73– 0.96)^‡^	0.88 (0.79–0.99)^‡^	1.00	<0.05
Total cholesterol	1.34 (1.13–1.60)^†^	1.10 (0.98–1.25)	1.11 (1.01–1.23)^‡^	1.00	0.09
Alcohol intake	0.98 (0.78–1.21)	0.99 (0.86–1.14)	1.05 (0.94–1.17)	1.00	0.45
Education	1.00 (0.81–1.23)	0.81 (0.71–0.93)^†^	1.09 (0.97–1.22)	1.00	0.68

All traits were drawn from the baseline examination and simultaneously included in a multinomial logistic regression model with the age of hypertension onset category as the dependent variable. *N* = 3,286. Odds for BMI, alcohol intake, total cholesterol, HDL-cholesterol, sedentary time, and education years are reported per 1-SD increase. The variable standard deviations are presented in Supplementary Table S1 online. A trend test was performed to examine a linear trend in odds ratios across hypertension onset age categories. Abbreviations: BMI, body mass index; HDL, high-density lipoprotein; HTN, hypertension; OR, odds ratio.

*P < 0.001.

^†^ *P* < 0.01.

^‡^ *P* < 0.05.

## DISCUSSION

Here, we demonstrate that black race, higher BMI, higher total cholesterol, and lower HDL-cholesterol level are related to increased odds early-onset hypertension. In particular, the odds for having earlier hypertension onset increased linearly across age of onset categories in black individuals and individuals with lower HDL-cholesterol. A few prior studies have reported shared characteristics among individuals with early-onset hypertension.^4,5,8^ These studies have reported male sex, black race, genetic background, higher serum triglyceride level, and high BMI as potential risk factors for early-onset hypertension. However, these studies were limited by a small study sample restricted to only patients with early-onset hypertension,^4,5^ or focused on finding optimal blood pressure thresholds for investigating secondary causes in early-onset hypertension.^8^ Blacks have previously been demonstrated to have a higher incidence of hypertension by 55 years of age among CARDIA study participants.^9^ In that study, black participants also had a 1.5- to 2.0-fold higher risk of developing hypertension throughout the follow-up compared with white individuals. However, here the hazard ratios were not separately reported for early- and late-onset hypertension. Our findings add higher total serum cholesterol and lower serum HDL-cholesterol as potential novel risk factors, and further verify black race and obesity as key risk factors for having early-onset hypertension. Additionally, our results provide information about the simultaneous relation of various risk factors for individuals with early hypertension onset age.

The strengths of our study include a large and diverse study sample drawn from the general population. Even though our study sample did not include elderly individuals, our study sample covers a larger age-spectrum than most previous studies which have investigated characteristics related to early-onset hypertension. Additionally, the study participants have been thoroughly followed up from early adulthood, enabling reliable assessment of hypertension onset age. As a limitation of this study, we had no information on parental hypertension, which could have impacted the results.

In our study, we identified black race, higher BMI, higher total cholesterol, and lower HDL-cholesterol as the strongest correlates of early-onset hypertension. Particularly black race and lower HDL-cholesterol level were associated with a trend toward earlier hypertension onset. Although many of these traits are established risk factors for hypertension, our study demonstrates that these features are particularly strongly associated with early-onset hypertension. Considering previous evidence on the excess cardiovascular disease risk of early-onset hypertension,^1–3^ our findings promote the need for more intensive hypertension screening and preventive measures in individuals who are black, obese, or have dyslipidemias. These individuals are at an increased risk of early-onset hypertension, a harbinger of cardiovascular disease.

## Supplementary Material

hpab066_suppl_Supplementary_Materials
