# Supplementary material for: Clinical Correlates of Early-Onset Hypertension
Source: Am J Hypertens. 2021 Apr 27;34(9):915–8. doi: 10.1093/ajh/hpab066 (PMC8457426; doi:10.1093/ajh/hpab066)
Supplement: hpab066_suppl_Supplementary_Materials [file hpab066_suppl_supplementary_materials.doc]

**Supplementary Information**

**Title:** Clinical Correlates of Early-Onset Hypertension

**Authors:** Karri Suvila MD,^a^ Joao A.C. Lima,^b^ Susan Cheng MD, MPH,^c,d,e^ Teemu J. Niiranen MD^a,f^

**Affiliations:** a) Division of Medicine, Turku University Hospital and University of Turku, Turku, Finland; b) Division of Cardiology, Johns Hopkins University, Baltimore, Maryland; c) Division of Cardiovascular Medicine, Department of Medicine, Brigham and Women's Hospital, Boston, USA; d) Smidt Heart Institute, Cedars-Sinai Medical Center, Los Angeles, CA, USA; e) Framingham Heart Study, Framingham, MA, USA; f) Department of Public Health Solutions, Finnish Institute for Health and Welfare, Turku, Finland

**Author Responsible for Correspondence:** Karri Suvila, address: Department of Internal Medicine, Kiinamyllynkatu 4–8, 20014 University of Turku, Finland; email: kahesuv@utu.fi; telephone/fax: +358 2 313 0000

**Running head:** Correlates of Early-Onset Hypertension

**Supplementary Table 1.** Baseline characteristics of the study sample and by age of hypertension onset categories.

|  |  | | Hypertension Onset Age | | | |  | |  |
| --- | --- | --- | --- | --- | --- | --- | --- | --- | --- |
| Characteristic | All | < 35 years | | 35–44 years | ≥45 years | No hypertension | | p-value |  |
| N | 3286 | 122 | | 332 | 486 | 2346 | | - |  |
| Age, years | 25.0 (3.6) | 25.1 (3.5) | | 24.3 (3.7) | 26.6 (3.0) | 24.8 (3.6) | | <0.001 |  |
| Women, n (%) | 1873 (57) | 62 (51) | | 195 (59) | 281 (58) | 1335 (57) | | 0.48 |  |
| Black race, n (%) | 1579 (48) | 95 (78) | | 249 (75) | 296 (61) | 939 (40) | | <0.001 |  |
| Body mass index, kg/m² | 20.7 (4.0) | 22.9 (4.9) | | 22.3 (4.8) | 21.8 (4.5) | 20.1 (3.5) | | <0.001 |  |
| Current smoker, n (%) | 854 (26) | 37 (30) | | 87 (26) | 157 (32) | 573 (24) | | <0.01 |  |
| Total cholesterol, mmol/l | 4.6 (0.9) | 4.8 (1.1) | | 4.7 (0.9) | 4.7 (0.9) | 4.5 (0.8) | | <0.001 |  |
| HDL-cholesterol, mmol/l | 1.4 (0.3) | 1.3 (0.3) | | 1.4 (0.3) | 1.4 (0.4) | 1.4 (0.3) | | <0.001 |  |
| Alcohol intake, ml/day (median, IQR) | 4.8 (0–14.5) | 2.4 (0–14.3) | | 2.7 (0–11.9) | 5.1 (0–15.2) | 4.8 (0–14.7) | | 0.42 |  |
| Education, years | 13.8 (1.8) | 13.4 (1.8) | | 13.2 (1.6) | 13.7 (1.8) | 13.9 (1.8) | | <0.001 |  |
| Systolic blood pressure, mmHg | 110 (11) | 123 (12) | | 114 (9.7) | 112 (10) | 108 (9.8) | | <0.001 |  |
| Diastolic blood pressure, mmHg | 68 (9.0) | 80 (9.9) | | 72 (8.8) | 69 (9.4) | 67 (8.2) | | <0.001 |  |

All values are reported as mean (SD) unless stated otherwise. Characteristics were obtained from the baseline exam. P-value indicating for any differences between hypertension onset age groups. IQR, interquartile range.
